# Cow activity measurements can be used to define new fertility traits for use in genetic evaluation

**DOI:** 10.3168/jdsc.2022-0251

**Published:** 2022-08-06

**Authors:** B. Heringstad, K.B. Wethal

**Affiliations:** 1 Department of Animal and Aquacultural Sciences, Faculty of Biosciences, Norwegian University of Life Sciences, PO Box 5003, 1432 Ås, Norway; 2 Geno Breeding and AI Association, Storhamargata 44, 2317 Hamar, Norway

## Abstract

Cow activity measurements are widely used in herd management tools for estrus detection. The aim of this study was to examine whether a new trait based on activity measures can be useful for genetic evaluation of cow fertility in Norwegian Red dairy cattle. Data from 284 herds with Lely milking robot were collected. Daily measurements of cow activity level were available from Lely activity tags and included data from 13,224 lactations of 8,139 Norwegian Red cows. We analyzed daily activity and included records from 10 to 150 d in milk for cows with at least 50 records. The trait analyzed was interval from calving to first high activity (CFHA). In total 87% of the cows had at least one episode of high activity recorded. The mean (standard deviation) of CFHA was 42 (28) days. A linear animal repeatability model with fixed effects of month-year of calving, age-parity, and herd, and random animal and permanent environment effects was used for estimation of variance components. The trait CFHA showed significant genetic variation with a heritability of 0.05 (0.01). This trait reflects the cow's ability to return to estrus cycle and show estrus after calving, which are important aspects of cow fertility.

## Highlights

• Activity tags measure cows' activity level and indicate heat.
• Cow activity measurements can be new fertility phenotypes.
• Interval from calving to first high activity showed significant genetic variation.
• New cow activity traits can supplement or replace current fertility traits.

## Graphical Abstract

**Figure:**
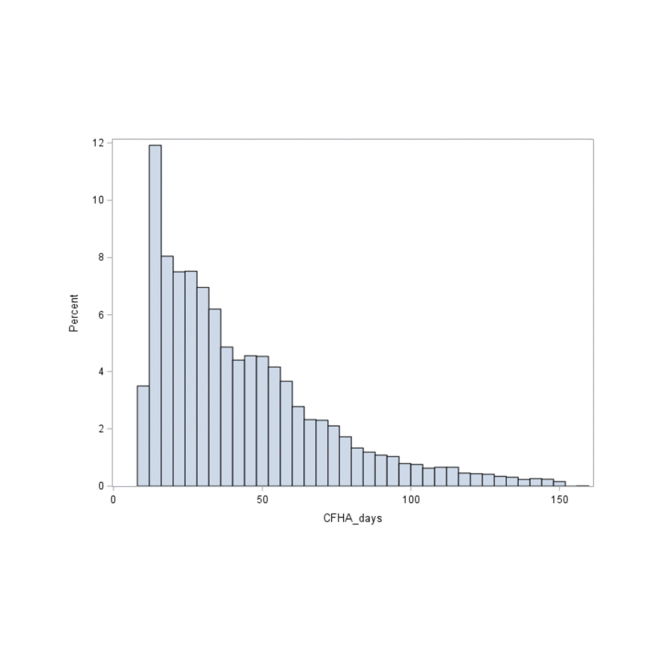
**Summary:** The aim of this study was to examine whether a new trait based on cow activity measures can be useful for genetic evaluation of cow fertility for Norwegian Red. Daily measurements of cow activity level were collected from 284 herds with Lely activity tags. We used records from 10 to 150 days in milk for cows with at least 50 records. After edits we had data from 13,224 lactations of 8,139 Norwegian Red cows. The trait analyzed was interval from calving to first high activity (CFHA). The mean (standard deviation) of CFHA was 42 (28) days. A linear animal repeatability model was used for estimation of variance components. The trait CFHA showed significant genetic variation with a heritability (standard error) of 0.05 (0.01). This trait reflects the cow's ability to return to estrus cycle and show estrus after calving, which are important aspects of cow fertility.

Cow fertility is a complex trait. Successful reproduction is the result of many factors such as early return to estrous cyclicity after calving, clear estrus behavior that enables timely insemination, high conception rate, implantation and survival of the embryo and fetus, an easy calving, and a vital calf. All of these interacting parts are affected by genetic as well as environmental factors (e.g., Lucy, 2019).

Breeding for improved cow fertility is possible and fertility has been included in Norwegian Red's breeding goal since the 1970s (Andersen-Ranberg et al., 2005). For many years Nordic countries were the only ones considering fertility traits in dairy cattle breeding. However, these traits have gained increased interest and emphasis in more recent years (Miglior et al., 2017; Fleming et al., 2019), and cow fertility is currently included in most national selection indices for dairy cows globally. Interbull started routine international genetic evaluation of fertility for Holstein in 2007 (www.interbull.org).

Current traits used for genetic evaluation of cow fertility are mainly derived from insemination dates and calving dates (Miglior et al., 2017), and include both success rate traits such as conception rate and nonreturn to first service, and interval traits such as interval from calving to first insemination, calving interval, and days open. These fertility phenotypes calculated from calving and inseminations dates can be largely affected by herd management decisions; thus, heritabilities are in general low. Better and more precise phenotypes that could be used to improve genetic evaluation of female fertility would therefore be of great value. New technology, for instance automatic estrus detection systems, offers new opportunities because they are widely used as herd management tools. Previous studies reported higher heritability for interval from calving to first high activity (**CFHA**), based on data from estrus detection systems, than for the traditional phenotype interval from calving to first insemination (**CFI**) (Løvendahl and Chagunda, 2009; Ismael et al., 2015). We wanted to examine whether new traits based on cow activity measures from commercial herds can be used for genetic evaluation of cow fertility in Norwegian Red, and the aim of this study was to estimate heritability of CFHA, a trait that reflects the cow's ability to return to estrus cyclicity and to show estrus after calving by increased level of activity.

Data were collected from 284 Norwegian herds with Lely (Lely Headquarters, Maassluis, the Netherlands) milking robot and activity tags. Activity measurements from 13,224 lactations of 8,139 Norwegian Red cows from the years 2016 to 2019 were included. Daily activity measures were available, and we included records from 10 to 150 DIM for cows with at least 50 records. Additional information about the cows, such as calving dates and pedigree, was available from the Norwegian Dairy Herd Recording System. High activity was defined using the variable “heat probability” generated in the herd management software. The trait analyzed was CFHA (no. of days). A total of 87% of the cows had at least one episode of high activity recorded. The mean CFHA was 42 d (SD = 28). No animals were used in this study, and ethical approval for the use of animals was thus deemed unnecessary.

A linear animal repeatability model was used for estimation of variance components with the AI-REML procedure in the DMU software (Madsen and Jensen, 2013). The model had fixed effects of month-year of calving, herd, and age-parity, and random effects of animal and permanent environment. The pedigree file included 83,022 animals.

The estimated additive genetic, permanent environment and residual variances for CFHA were 32.14, 13.35, and 596.15, respectively, with corresponding heritability (SE) of 0.05 (0.01). Previous studies reported higher heritability for CFHA. Løvendahl and Chagunda (2009) estimated a heritability of 0.18 (0.07) for the trait days to first high activity based on activity tag data from 517 cows in a Danish experimental herd. Ismael et al. (2015) had 3,533 Danish Holstein cows with phenotypic records of CFHA and estimated a heritability of 0.16 (0.04), whereas Ismael et al. (2017) estimated a heritability of 0.14 (0.01) for CFHA based on phenotypes from 25,733 Holstein cows. Whereas both Løvendahl and Chagunda (2009) and Ismael et al. (2015) reported higher heritability for CFHA than for CFI, our estimated heritability of CFHA was in line with Andersen-Ranberg et al. (2005) who estimated a heritability of 0.03 for CFI of Norwegian Red.

Better and more precise phenotypes for fertility can contribute to more accurate genetic evaluations. Automatic, continuous recording of cow activity may provide objective and frequent measures of traits related to some aspects of cow fertility. Traits derived from activity measures are less affected by herd management decisions than traditional fertility traits based on insemination dates. Another advantage is that records may be available for all cows, including those that never get inseminated. Cow activity measures are widely used as a tool for estrus detection, and records already exist for a large proportion of the population. However, there are still challenges related to access data on a regular basis. Many different providers, systems, and versions are in use, and a system for routine collection of data needs to be established before activity data can be used in routine genetic evaluations of cow fertility.

Our results show that cow activity measurements can be used for genetic evaluations. The trait CFHA showed significant genetic variation. The CFHA reflects the cow's ability to return to estrus cyclicity and to show estrus after calving by increased level of activity. Both are important aspects of cow fertility. Measures of cow activity provide information on new traits that can supplement or replace current fertility traits.
